# Impact of conflict on the elimination targets of lymphatic filariasis, schistosomiasis and soil-transmitted helminths in Cabo Delgado province, Mozambique

**DOI:** 10.1371/journal.pntd.0012119

**Published:** 2024-04-18

**Authors:** Xavier Badia-Rius, Henis Mior Sitoe, Sergio Lopes, Louise A. Kelly-Hope

**Affiliations:** 1 The MENTOR Initiative, Haywards Heath, United Kingdom; 2 Direcção Nacional de Saúde Pública, Ministério da Saúde, Maputo, Mozambique; 3 Institute of Infection, Veterinary & Ecological Sciences, University of Liverpool, United Kingdom; Universidade do Estado do Rio de Janeiro, BRAZIL

## Abstract

**Background:**

Mozambique has one of the highest burdens of neglected tropical diseases in Africa. Lymphatic filariasis, schistosomiasis and soil-transmitted helminths are being targeted for elimination as part of integrated mass drug administration campaigns. The progress made towards interruption of transmission has been affected by recent conflict in Cabo Delgado province. The aim of this paper was to determine the potential impact of this crisis on the neglected tropical diseases programme and the challenges in reaching the elimination goals of 2030.

**Methodology:**

A desk-based secondary data analysis was conducted on publicly available sources of neglected tropical diseases, conflict incidents, internally displaced persons and geographical access between 2020 and 2022. Data were summarised and mapped using GIS software. A combined risk stratified assessment at district level was developed with five classifications i) Very high-risk; ii) High-risk; iii) Medium to high-risk; iv) Medium risk; and v) Not at risk due to conflict absence but co-endemic.

**Results:**

Lymphatic filariasis, schistosomiasis and soil-transmitted helminths were co-endemic in 115 out of 156 (74%) districts. Between 2020 and 2022 a total of 1,653 conflict-related incidents were reported, most of them in Cabo Delgado province (n = 1,397, 85%). A five-fold increase of internally displaced persons was recorded from April 2020 (n = 172,186) to November 2022 (n = 935,130). Geographical accessibility also deteriorated across the province with an increase from five (29%) in 2021 to seven (41%) districts in 2022 classified as hard-to-reach. The combined risk stratification identified that most districts in Cabo Delgado province had medium to high-risk (n = 7; 41%); very high-risk (n = 5, 29%); medium risk (n = 3, 18%); high-risk (n = 2, 12%).

**Conclusion:**

Most of the districts of Cabo Delgado were considered to be at risk of not meeting the neglected tropical diseases road map 2030 targets due to the humanitarian crisis ongoing. There is the need for practical strategies and funding to overcome these hostile challenges.

## Author summary

Neglected tropical diseases (NTDs) are a group of diseases that usually affect the most vulnerable population in tropical countries. Mozambique has been tackling lymphatic filariasis, schistosomiasis and soil-transmitted helminths for two decades with the aim of eliminating them through large-scale distribution of drugs to interrupt transmission. In recent years, internal conflict has emerged and led to an escalating humanitarian crisis in the endemic province of Cabo Delgado which has hampered elimination efforts. This study aimed to shed light on the situation and to determine the potential impact on the Mozambique NTDs’ programme.

We used a range of open-source data on NTDs, conflict, internally displaced persons and geographical access to summarise, describe, and map the situation at the district level in the most affected province. We used this data to also examine their geographical overlap and to develop a risk assessment tool to highlight the high risk and most vulnerable areas. We found that between 2020–2022 more than 1,300 conflict incidents occurred, and almost 1 million people were displaced in Cabo Delgado province with many districts having limited geographical access which impacted the NTDs programme’s ability to work.

The study highlights the important challenges of conflict and displacement of vulnerable populations, and the need for support and set of guidelines for NTDs endemic programmes to use to help to overcome key barriers and meet the targets of elimination set by the World Health Organization for 2030.

## Introduction

Neglected tropical diseases (NTDs) are a group of 20 conditions that affect mainly impoverished communities in tropical regions of the world [1]. The World Health Organization (WHO) recommends Mass Drug Administration (MDA) campaigns as the primary public health intervention strategy to eliminate the five Preventive Chemotherapy (PC) NTDs: lymphatic filariasis, onchocerciasis, schistosomiasis, soil-transmitted helminths and trachoma [2,3]. The largest burden of these diseases is in sub-Saharan Africa where 38 countries are currently endemic for at least one of these PC NTDs [4].

In 2020, the WHO released the road map for NTDs 2021–2030, which sets global targets towards the control, elimination, or eradication for all listed NTDs [5]. The NTD road map also sets cross-cutting objectives and highlights numerous challenges that national programmes currently face. Conflict and violence, and population movement/displacement were identified as major challenges, yet little is known about their impact on NTD programmes and how it may affect disease transmission dynamics. Currently, there are no global or national guidelines to address these issues, which will hinder many conflict-prone countries’ ability to meet the NTD road map targets [6]. In addition, there is little published evidence about the impact of displacement in NTD programmes and evidence is lacking on how to ensure continued NTD interventions in conflict affected areas.

Mozambique is a large country in south-eastern Africa with a high burden of PC NTDs. In recent years, there has been a surge in conflict events [7], which has negatively impacted its populations and the NTD programme’s operational capacity. In Mozambique, lymphatic filariasis, schistosomiasis and soil-transmitted helminths are widely prevalent with various levels of co-endemicity requiring coordinated integrated MDA campaigns including the donated drugs ivermectin (for lymphatic filariasis), albendazole (for lymphatic filariasis and soil-transmitted helminths) and praziquantel (for schistosomiasis). Lymphatic filariasis is endemic in 114 (71%) districts, while schistosomiasis and soil-transmitted helminths endemicity extends to all 161 (100%) districts [4, 8]. The endemicity status of onchocerciasis is still unknown in 135 (84%) districts while the rest are classified as non-endemic. Trachoma is endemic in 65 (40%) districts and requires separate MDA activities [4,9].

Mozambique PC NTD programmes were initiated at different times between 2005–2010. Overall, there had been steady progress in scaling up MDA across the country with national and international technical and financial support [4]. By 2016, positive steps had been made towards lymphatic filariasis elimination, with several districts in Niassa and Tete provinces achieving the post-treatment surveillance phase, and activities for the morbidity management and disability prevention were being started and integrated into health systems. There is a lack of data and knowledge on onchocerciasis endemicity with the necessity to conduct mapping in most of the country. Less is known about the progress of schistosomiasis and soil-transmitted helminths control as few epidemiological monitoring surveys have been conducted, with the last sentinel site survey completed in 2015. Trachoma progress towards elimination has been remarkable as 46 districts were under post-MDA surveillance in 2021 [4].

Unfortunately, in 2017 this progress was disrupted when the province of Cabo Delgado in northern Mozambique started to experience a humanitarian crisis as a consequence of conflict and violence perpetrated by Organised Armed Groups [10]. Serious events have been reported such as the attack on the town of Palma in March 2021 with dozens of casualties and more than 30,000 displaced [11]. The crisis has caused the forced displacement of more than one million people. Further, many programme activities have had to stop or be curtailed due to restricted geographical access to the districts affected, as well as a change in the level of external funding. Additionally, many health facilities have been damaged thereby limiting their functionality as focal NTD points and centres of clinical care [12].

The increasing threat of conflict and displacement on NTD programmes has started to be examined [13–15]. However, more still needs to be addressed as it is a neglected area that will hinder programme progress and success. The aim of this paper was to build on this recent work and draw attention to the humanitarian crisis in Cabo Delgado province and the impact and implications for the Mozambique NTD programme. Specifically, it focused on lymphatic filariasis, schistosomiasis and soil-transmitted helminths, and it examined their co-endemicity in relation to conflict incidents, internally displaced persons (IDPs), and geographical accessibility across the endemic districts. Further it developed a practical stratified assessment method taking the different levels of risk into account to better understand the complex challenges, identify potential solutions, and provide insights on the risk of not reaching the NTD road map targets of 2021–2030.

## Methods

### Ethics statement

The data included in this study were freely available in publicly open data sources and therefore no ethics was required for the secondary data analysis presented in this paper.

### Study design and population

This observational study was conducted through a desk-based secondary data analysis compiling a range of publicly available data of NTD co-endemicity, conflict-related incident events, IDP presence, and geographical accessibility in Mozambique from 2020 to 2022. These data were examined together and used to create a series of stratified maps detailing different levels of risk.

### Data sources and analysis

#### Neglected tropical diseases

The NTD data per district were obtained from the WHO Expanded Special Project for Elimination of Neglected Tropical Diseases (ESPEN) data portal [4]. The focus was on three PC NTDs that are treated through an integrated MDA platform: lymphatic filariasis, schistosomiasis, and soil-transmitted helminths. The Joint Request for Selected PC Medicines (JRSM) file updated on the 30 January 2023, was used to summarise and map the level of co-endemicity.

#### Conflict incidents

Conflict and violence-related incident data were obtained from The Armed Conflict Location & Event Data Project (ACLED) data export tool [16]. The data covered various type of events including battles (e.g., armed clash); protests (e.g., protest with intervention,); riots (e.g., violent demonstration); explosions/remote violence (e.g., artillery attack); violence against civilians (e.g., sexual violence, attack); and strategic developments (e.g., arrests, looting). All ACLED incidents recorded between 2020 and 2022 in Mozambique were exported and summarised in Microsoft Excel [17]. Maps of Mozambique and Cabo Delgado province were created to visualise the number and location of incidents over time.

#### Internally displaced persons

IDPs are persons or groups of persons who have been forced or obliged to flee or to leave their homes or places of habitual residence, in particular as a result of or in order to avoid the effects of armed conflict, situations of generalised violence, violations of human rights or natural or human-made disasters, and who have not crossed an internationally recognised border. The difference with a refugee is that the latter crosses an international border [18]. The number of IDPs in each endemic district were obtained from the International Organization for Migration (IOM) Displacement Tracking Matrix (DTM) portal [19]. Mozambique DTM assessments conducted between 2020 and 2022 were used to summarise IDP numbers per district at different time points: December 2020, November 2021 and November 2022. Maps of Cabo Delgado province were created to visualise the IDP numbers over time.

#### Geographical accessibility

Information on the level of geographical access was based on the United Nations Office for the Coordination of Humanitarian Affairs (OCHA) monthly access maps between January 2021 and December 2022, available from the humanitarian information website ReliefWeb [20]. OCHA classified each district as:

i) Accessible
ii) Partially accessible: physical, logistical, or administrative constraints may impact operations. Security environment difficult to predict; humanitarian operations will require security planning and negotiations,
iii) Hard-to-reach: Security situation highly volatile; humanitarian operations will require detailed security planning and intensive negotiations.

To account for the variability throughout the year, the most reported access level was used, e.g., in 2022, Chiure district was classified by OCHA as partially accessible during 7 out of 12 months, therefore, Chiure was classified as ‘partially accessible’ for that year. Annual summary maps were created for Cabo Delgado province to visualise the main level of access for each district in each year.

### Combined risk stratification

A stratified assessment method was developed and based on the combination of all data according to the different groupings of risk; conflict and violence incidents presence (i.e., incidents presence/absence), IDPs high or low presence (i.e., high classified as above 10,000 IDPs; low as <10,000 IDPs) and geographical accessibility (i.e., accessible, partially accessible or hard-to-reach).

The co-endemic districts of Cabo Delgado province were stratified into five categories including:

1. Very high-risk districts: hard-to-reach; high IDP presence;
2. High-risk districts: hard-to-reach; low IDP presence;
3. Medium to high-risk districts: accessible and partially accessible; high IDP presence;
4. Medium risk: accessible and partially accessible; low IDP presence;
5. Districts not at risk due to conflict absence but co-endemic.

All districts of Cabo Delgado were considered to have been affected by the conflict due to the register of attacks in all districts and the inclusion of the districts on the OCHA accessibility assessment maps. Other neighbouring districts outside Cabo Delgado were also affected by the conflict and some of them were included in the maps classifications. However, the analysis of the results only considered Cabo Delgado province.

All maps were created using QGIS 3.16.16 [21] with administrative boundaries from the Humanitarian Data Exchange (HDX) [22]. The variables used for the analysis were compiled in the CVS format and imported to QGIS to create the colour-coded maps highlighting the different variable strata and risk combinations.

## Results

### Neglected tropical diseases

**Fig 1:**
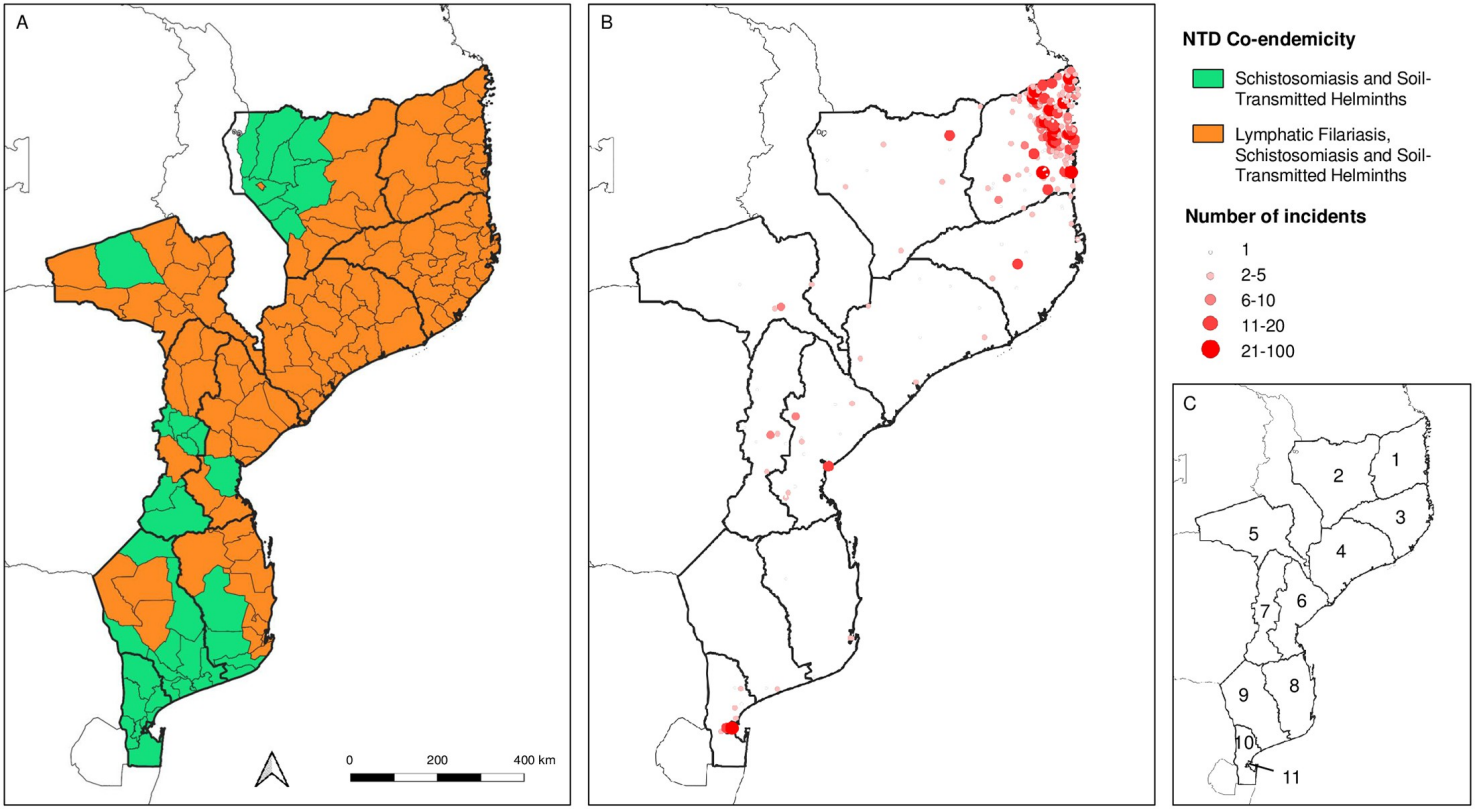
Overview of NTD co-endemicity and recorded conflict incidents between 2020 and 2022 by province. (A) Co-endemicity of lymphatic filariasis, schistosomiasis and soil-transmitted helminths. (B) Incidents recorded in Mozambique between 2020 and 2022. (C) Provinces of Mozambique: (1) Cabo Delgado, (2) Niassa, (3) Nampula, (4) Zambezia, (5) Tete, (6) Sofala, (7) Manica, (8) Inhambane, (9) Gaza, (10) Maputo Province, (11) Maputo City. Source: ESPEN (https://espen.afro.who.int/); ACLED (https://acleddata.com/); administrative boundaries from Humanitarian Data Exchange (https://data.humdata.org/dataset/cod-ab-ago).

For schistosomiasis and soil-transmitted helminths, all 156 (100%) districts of Mozambique were classified as endemic. For lymphatic filariasis, 115 (74%) districts were classified as endemic (Fig 1A). In total 115 (74%) districts were co-endemic with lymphatic filariasis, schistosomiasis and soil-transmitted helminths.

### Conflict

In total, 1,653 conflict-related incidents were recorded in the ACLED database; in 2020 (n = 459), 2021 (n = 410) and 2022 (n = 528) (Fig 1B). These incidents included different types of events: battles (n = 446); protests (n = 71); riots (n = 60); explosions/remote violence (n = 57); violence against civilians (n = 787); and strategic developments (n = 232).

The highest number of incidents were reported in Cabo Delgado province with a total of 1,397 incidents (84.5% of total) and 3,510 fatalities (96.6% of total). The type and number of incidents included violence against civilians (n = 698), battles (n = 429), strategic developments (n = 192), explosions/remote violence (n = 56), riots (n = 16) and protests (n = 6).

The combination of NTDs co-endemicity map and incidents map in Fig 1 shows that the northern provinces of Mozambique are the ones with higher burden of NTDs and especially Cabo Delgado has been impacted the most with the current conflict.

**Fig 2:**
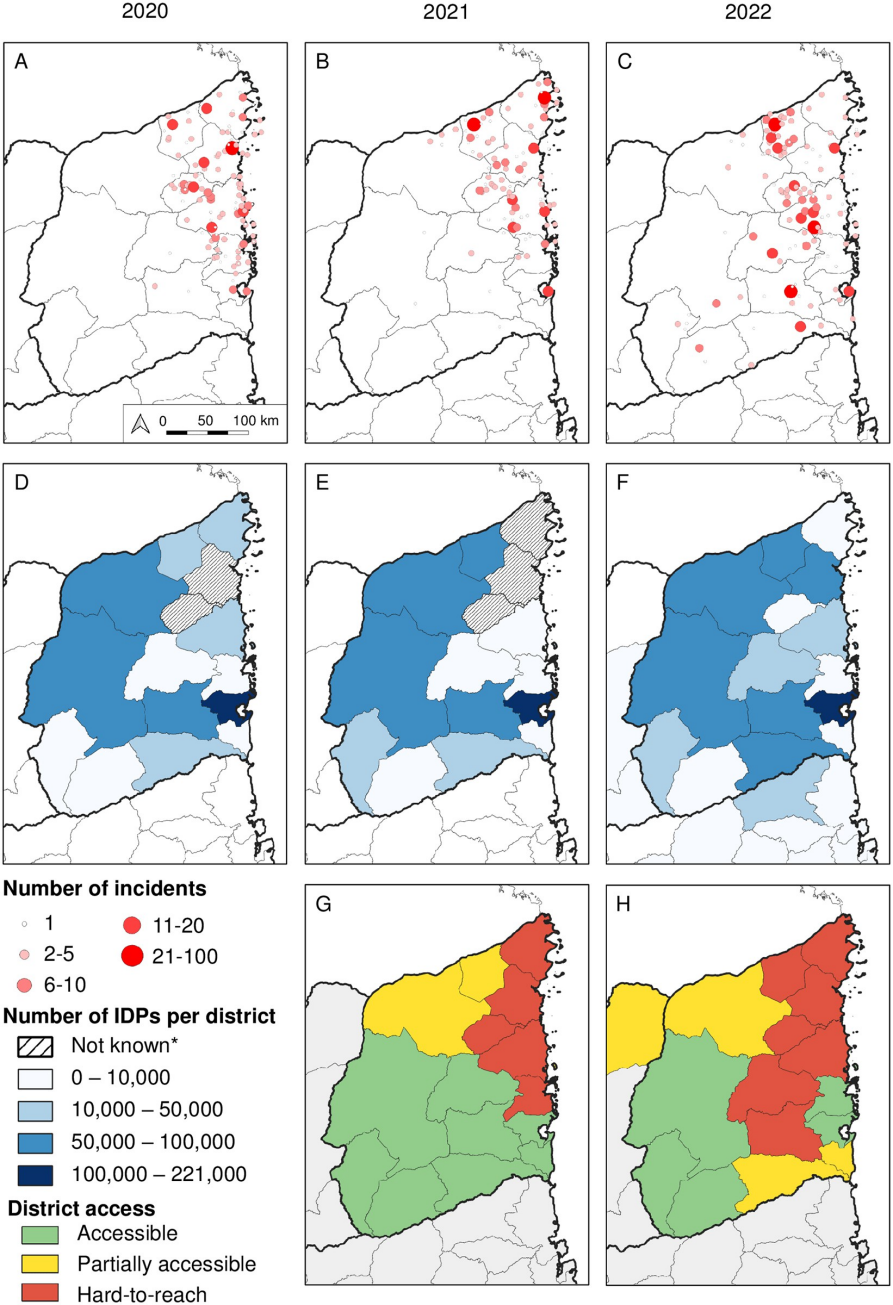
Overview of incidents, Internally Displaced Persons and accessibility in Cabo Delgado province 2020–2022. (A) Incidents recorded in Cabo Delgado in 2020, (B) 2021 and (C) 2022. (D) Internally Displaced Persons per district in December 2020, (E) November 2021 and (F) November 2022. (G) Area access in 2021 and (H) 2022. *Not known due to lack of access. Source: ACLED, IOM DTM, OCHA, administrative boundaries from Humanitarian Data Exchange (https://data.humdata.org/dataset/cod-ab-ago).

The maps in Fig 2A and 2B highlight that the incidents in Cabo Delgado province occurred in the northern and coastal districts in 2020 and 2021. However, in 2022 the incidents expanded to the southern and western districts of the province (Fig 2C).

### Internally displaced persons

The IOM April 2020 assessment recorded a total of 172,186 IDPs in Cabo Delgado province. This number significantly increased and by December 2020 the number reached 607,100 IDPs (Fig 2D). The IOM November 2021 assessment recorded a total of 663,276 IDPs in the province, which increased to 935,130 IDPs by November 2022 (Fig 2E and 2F).

### Geographical accessibility

**Fig 3:**
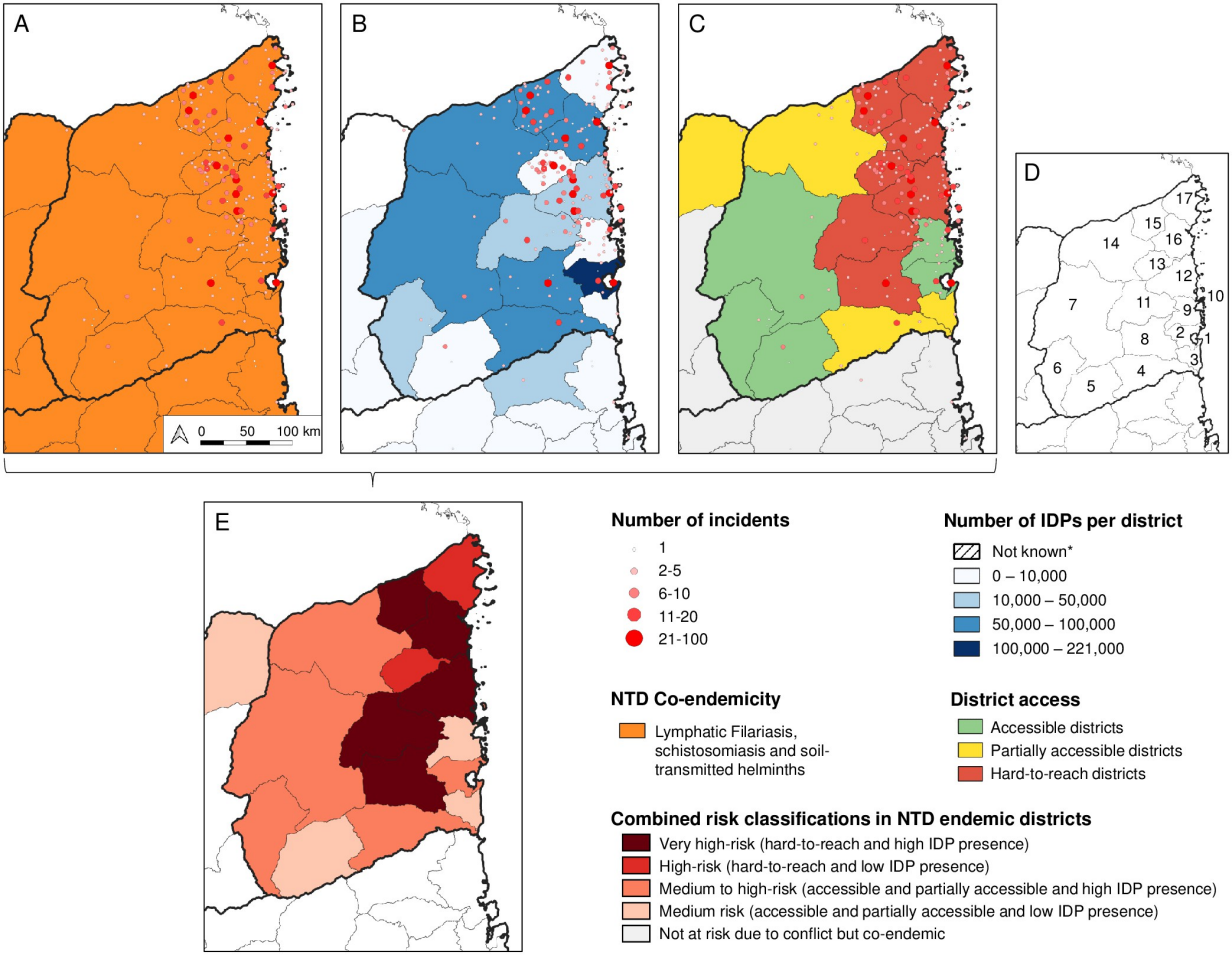
Combined risk maps of NTD co-endemicity, area accessibility due to conflict, Internally Displaced Persons (IDPs) presence and incidents. (A) Co-endemicity of lymphatic filariasis, schistosomiasis and soil-transmitted helminths and incidents recorded in Cabo Delgado in 2022. (B) IDP presence in November 2022 and incidents recorded in Cabo Delgado in 2022. (C) Accessibility due to conflict in 2022 and incidents recorded in Cabo Delgado in 2022. (D) Cabo Delgado Province and its districts: (1) Pemba, (2) Metuge, (3) Mecufi, (4) Chiure, (5) Namuno, (6) Balama, (7) Montepuez, (8) Ancuabe, (9) Quissanga, (10) Ibo, (11) Meluco, (12) Macomia, (13) Muidumbe, (14) Mueda, (15) Nangade, (16) Mocímboa da Praia, (17) Palma. (E) Combined risk map of NTD, Accessibility and IDP presence. Administrative boundaries from Humanitarian Data Exchange (https://data.humdata.org/dataset/cod-ab-ago).

Data for 2021 summarised in Fig 2G highlight 5 (29%) districts classified as hard-to-reach with high insecurity along the northern coastal region of the province, including Palma, Mocímboa da Praia, Muidumbe, Macomia and Quissanga (see Fig 3D for district identification). The northern districts of Nangade and Mueda as well as the islands of Ibo were classified as partially accessible.

Fig 2H highlights the increase in the number of districts classified as hard-to-reach in 2022 (n = 7, 41%), adding Nangade, Meluco and Ancuabe. The southern districts of Chiure and Mecufi were classified as partially accessible. Quissanga district classification changed from being hard-to-reach to being accessible.

### Risk stratification

The stratified maps based on combined data are shown in Fig 3. Fig 3A highlights the lymphatic filariasis, schistosomiasis and soil-transmitted helminths co-endemic districts and the conflict incidents recorded in Cabo Delgado province in 2022.

Fig 3B shows the geographical overlap between the conflict incidents and number of IDPs in 2022. Two districts had more than 100,000 IDPs (incidents n = 18; mean = 9), 6 districts had between 50,000 and 100,000 IDPs (n = 253; mean = 42.2), 4 districts had between 10,000 and 50,000 IDPs (n = 166; mean = 41.5), 5 districts had less than 10,000 IDPs (n = 91; mean = 18.2).

Fig 3C shows the geographical overlap between the conflict incidents and levels of access in 2022. Out of the 17 districts in Cabo Delgado, 7 were hard-to-reach districts (incidents n = 296; mean = 62), 3 partially accessible districts (n = 35; mean = 11.7), 7 accessible districts (n = 59; mean = 8.4).

Fig 3E shows the combined risks classifications in the endemic districts. In total, 5 districts were classified as very high-risk, 2 districts as high-risk, 5 districts as medium to high-risk, and 4 districts as medium risk. Nangade, Mocímboa da Praia, Macomia, Meluco and Ancuabe districts had a very high-risk classification due to being hard-to-reach areas with high IDP presence. Palma and Muidumbe were classified as high-risk due to being hard-to-reach with low IDP presence. Mueda, Montepuez, Balama, Chiure, Metuge, Ibo and Pemba had a medium to high-risk due to being accessible and partially accessible with high numbers of IDPs. The remaining districts, Quissanga, Mecufi and Namumo in Cabo Delgado province as well as the neighbouring Mecula district of Niassa province were classified as medium risk because of the low presence of IDP and being accessible or partially accessible.

## Discussion

This paper sheds light on the conflict-related humanitarian crisis, its repercussions and the challenges facing the NTD programme in Mozambique. It underscores the potential setbacks in achieving the NTD road map 2021–2030 targets related to the elimination of lymphatic filariasis, schistosomiasis and soil-transmitted helminths, particularly in the Cabo Delgado province. It focuses on one set of NTDs that are targeted with an integrated drug intervention, but the impact and implications may extend to all NTDs and other national programmes addressing endemic diseases. The development of a stratified assessment method, taking different levels of risk of conflict, IDPs and geographical access into account, builds on other recent approaches [13–15] and will help the NTD programme better address the specific challenges of each district within Cabo Delgado province.

The escalation of armed conflict, coupled with mass forced population movements, and limited accessibility for the local populations and IDPs has impeded a range of NTD activities, including for example, MDA campaigns, morbidity and disability management, and impact assessments such as sentinel site surveys and Transmission Assessment Surveys [4]. These delays have placed Cabo Delgado province at a disadvantage, falling behind the less affected neighbouring Nampula and Zambezia provinces, which had similar baseline prevalence. The potential consequence of these disruptions on disease transmission is uncertain, and it is possible that if activities are not resumed soon, there is a risk of resurgence and/or (re)introduction to new and/or low endemic areas, which will have significant logistical and cost implications similar to other complex emergency situations [23–25].

The conflict-related crisis in Mozambique, as well as in other parts of sub-Saharan Africa, highlights the need for specialised guidelines [13]. These guidelines should include modified survey methodologies tailored to the impact of conflict, the presence of IDPs, and populations in hard-to reach areas. Such modifications may help to assess the transmission status more accurately [13,26–28]. Furthermore, the crisis raises the need for increased funding to support these vulnerable populations, who are forced to move with limited access to health care in these extraordinary humanitarian situations [29]. In Mozambique, funding disruptions have affected all NTD programmes, potentially exacerbating disparities within the country. Further, the situation sets Mozambique apart from its neighbouring endemic countries that are making good progress, such as Malawi and Zambia [30].

The impact of internal displacement driven by conflict is of increasing concern in Africa, particularly. Displaced populations have dramatically increased in sub-Saharan Africa reaching 31.7 million IDPs in 2022 [31]. IDPs are at highest risk of communicable diseases due to overcrowding, malnutrition, and lack of access to hygiene and safe water. In addition, limited access to health services, food insecurity and disruption of livelihoods negatively impacts the resilience capacity of these communities [27]. Case studies have shown how higher incidence of human African trypanosomiasis and cutaneous leishmaniasis correspond to increases in the intensity of conflict [32]. The consequences of mass displacement due to conflict in the progress towards elimination of lymphatic filariasis, schistosomiasis and soil-transmitted helminths are still a significant gap to be studied.

Working with a variety of stakeholders from different sectors will help to develop the national capacity to respond more effectively to these complex emergencies. Valuable lessons may be learned from the humanitarian sector, offering potential collaborations, co-implementation opportunities, and insights on adapting programmes in humanitarian situations [33–35]. Moving forward, it will be important for the Mozambique NTD programme to comprehensively document the impact of this crisis on all its programmes as they may be affected differently. Additionally, a set of preferred practices, and the implementation of an advocacy and communication strategy will help to inform national and international stakeholders of the key threats and successes. Flexibility on operations and funding, social mobilisation, detailed and strict security protocols or negotiations for ceasefires have been reported as strategies to tackle some of the challenges these settings have [36,37].

The main limitation of this study is related to the publicly available data on NTDs, conflict, IDPs and geographical access. These data were sourced from different providers and had varying levels of spatial and temporal detail. Further, they may not have been complete or only relevant at the time of extraction, given the situation is constantly changing. Another limitation was the inability to use programmatic data to analyse the impact of the conflict in the implementation of the activities. Notwithstanding these limitations, the data were obtained from the best sources available in the public domain, and the combined use of these data is novel and draws attention to a neglected area that affects many conflict-prone endemic countries in Africa that have the potential risk of not meeting the NTD road map targets.

Further work on assessing and quantifying the impact of conflicts on the progress towards NTD elimination (delays on MDA, monitoring and evaluation surveys and morbidity management and disability prevention) in Mozambique and in other NTD affected countries will help to understand the magnitude of the problem to raise awareness and mobilise resources. Operational research is needed to understand the validity of the current monitoring and evaluation framework in displaced populations and to evaluate the effectiveness of different treatment methodologies used in these difficult settings [38]. Addressing NTDs in conflict settings is essential to meet control, elimination and eradication targets and not to leave anyone behind.

## Supporting information

S1 Text STROBE Statement. Checklist of items that should be included in reports of cross-sectional studies.
(DOCX)
